# Perfectionism Classes and Aggression in Adolescents

**DOI:** 10.3389/fpsyg.2021.686380

**Published:** 2021-06-01

**Authors:** Cecilia Ruiz-Esteban, Inmaculada Méndez, Aitana Fernández-Sogorb, José Daniel Álvarez Teruel

**Affiliations:** ^1^Department of Evolutionary and Educational Psychology, Faculty of Psychology, University of Murcia, Murcia, Spain; ^2^Department of Developmental Psychology and Didactics, Faculty of Education, University of Alicante, Alicante, Spain

**Keywords:** adolescents, perfectionistic strivings, perfectionistic concerns, latent class analysis, aggressive behavior

## Abstract

Some of the components of perfectionism produce a variety of problems, such as interpersonal hypersensitivity and hostility, that may be associated with aggression behavior during adolescence. This study aims to identify classes of adolescents depending on their levels of Perfectionistic Strivings (PS) and Perfectionistic Concerns (PC) as well as to examine whether there are significant differences in the manifestations of the four components of aggression behavior (i.e., anger, hostility, physical aggression, and verbal aggression) between them. A total of 1,074 high school students from various educational centers participated in this study (*M* = 14.78, *SD* = 1.84). The Child-Adolescent Perfectionism Scale and the Aggression Questionnaire short form were used. The Latent Class Analysis identified three classes of adolescent perfectionism: (a) Non-Perfectionists (low PS and PC), (b) Maladaptive Perfectionists (high PS and PC), and (c) Adaptive Perfectionists (moderate PS and PC). Results revealed significant differences between classes regarding the different manifestations of aggression. Maladaptive Perfectionists and Adaptive Perfectionists reported, respectively, the highest and lowest levels of aggression behavior. This study assists in educational programs to prevent conflicts related to school violence through emotional adjustment.

## Introduction

Perfectionism is a personality trait characterized by the imposition of extremely high-performance standards upon oneself that are unrealistic, along with the motivation to achieve perfection and the perception of one’s environment as too demanding and critical (García-Fernández et al., 2016). According to Flett et al. (2000, unpublished), child and adolescent perfectionism is constituted by the dimensions of Socially Prescribed Perfectionism, defined as the belief that people in the environment demand that you be perfect, and Self-Oriented Perfectionism, understood as the motivation to achieve perfection in the achievement of tasks along with high performance expectations. Thus, perfectionism is a multidimensional personality trait (Hewitt et al., 2008) with both, intrapersonal and interpersonal components.

The two-factor theory of perfectionism argues that it is possible to classify any perfectionist dimension into two higher-order dimensions: one is considered adaptive and it is called Perfectionistic Strivings (PS), while the other is considered maladaptive and is commonly called Perfectionistic Concerns (PC; Stoeber, 2018). PS involves rigid and unrealistic demand for self-perfection. There is a debate about the adaptive or maladaptive nature of PS. Some researchers point out that PS are positively related to psychopathology, especially as a risk factor for eating disorders (Bardone-Cone et al., 2007) or after a performance failure (Besser et al., 2004) or to hostility (Vicent et al., 2017). Although this debate continues, it is generally accepted that PS tend to be less related to negative outcomes than PC.

On the other hand, PC are a latent construct that includes the self-critical aspects of perfectionism, such as perception of the demand for perfection by others, doubts about personal competence, self-punishment for failure, and self-deception about personal performance. This latent construct represents a set of diverse constructs that include interpersonal personality (Hewitt and Flett, 1991), cognitive behavioral (Frost et al., 1990), psychodynamics (Blatt et al., 1976), and psychology of counseling (Slaney et al., 2001). PC constitutes a risk factor for mental health, such as depression, anxiety, eating disorders, autolysis, and personality disorders (Hewitt and Flett, 1991), as well as for physical health such as sleep disorders or sexual dysfunction (Stoeber et al., 2013). Also, people high in PC tend to experience many interpersonal problems (Habke and Flynn, 2002) such as loneliness, interpersonal conflicts, hostility, problems with perceived social support, divorce, etc. (Hewitt et al., 2008). Thus, most authors point out PC as a maladaptive construct (Sherry et al., 2016).

Thus, taking into account the diversity of scales and subscales of perfectionism that exists; this two-factor model allows compare previous research as different subscales can be employed as indicators of PS and PC. In this sense, Socially Prescribed Perfectionism, for example, is considered an indicator of PC, whereas Self-Oriented Perfectionism is employed as a measure of PS. Other authors, however, have used other dimensions as indicators of PS and PC (Frost et al., 1990).

### Perfectionism Profiles

Several authors have used different scales and subscales for identifying perfectionism profiles. However, in order to interpret the results of these studies under a common framework, the terms PS and PC will be used. Although different profile solutions have been found by previous research analyzing perfectionism profiles, most of studies using the methods of Latent Profile Analysis or Latent Class Analysis found a three-class solution. For example, Gilman et al. (2014) found three profiles of perfectionism in a study among 718 high school students from the United States: adaptive perfectionists (high PS and low PC) which represented a 61.8% of the sample, maladaptive perfectionists (high PS and PC) representing the 15.7% of the sample, and non-perfectionists (low PS and PC) classifying the 22.25% of the sample. Moate et al. (2016) also found a three-profile solution: adaptive perfectionists (high PS and low PC), maladaptive perfectionists (high PS and PC), and non-perfectionists (low PS and PC), who represented 58.1, 28.8, and 13.1%, respectively, in a sample of 528 doctoral students from the United States. In another study among 186 Russian undergraduate students, Wang et al. (2016) found the same profiles than Moate et al. (2016) with a prevalence of 39, 34 and 27%, respectively. Similarly, Vicent et al. (2019b), in a sample of 431 Spanish primary school students, found three profiles of perfectionism: high perfectionism (high PS and PC), moderate perfectionism (moderate PS and PC), and non-perfectionism (low PS and PC) representing, respectively, the 26.68, 62.41 and 10.90% of the sample.

### Perfectionism Profiles and Aggression Behavior

The first and unique study by Vicent et al. (2017) has analyzed the relationship between perfectionism and the four components of aggression behavior from a person-oriented approach. Thus, in a sample of 1,815 Spanish students between 8 and 11 years, four profiles of child perfectionism through a cluster analysis were identified: Non-Perfectionism (low PS and PC), Pure PC (low PS and high PC), Pure PS (moderate PS and low PC), and Mixed Perfectionism (high PS and PC). In terms of aggression, Mixed Perfectionism was the most maladaptive profile reporting the highest scores on the four factors of aggression behavior as well as on the total score. In contrast, Non-Perfectionism and Pure PS were more adaptive than the others were as they reported the lowest scores on aggression behavior. However, although results of Vicent et al. (2017) provided evidence about the relationship between child perfectionism profiles and aggression behavior, the study has several limitations. First, results of this study cannot be generalized to other samples such as adolescents or adults. Since, according to the review prepared by Sánchez de la Flor (2018), aggressive behavior changes with age. Aggression in the first years of life is instrumental and evolves in a stable way until 5–6 years when the child shows a more reactive type of aggression (hostile and verbal). Preadolescence is key in the development of aggressiveness, being the age most vulnerable to the development of this type of behavior, decreasing these as the subject grows. Secondly, the method employed, specifically a non-hierarchical cluster analysis presents several limitations, which has been overcome by other techniques such as Latent Class Analysis (LCA). In fact, LCA is considered nowadays a more appropriate method for researching about profiles (Schreiber, 2017).

### This Study

This study tries to overcome the limitations of Vicent et al. (2017) work mentioned above by analyzing the relationship between perfectionism and the four components of aggression behavior from a person-oriented approach in a sample of Spanish adolescents. Specifically, it is aimed (a) to identify profiles of different combinations of the perfectionist dimensions, PS and PC, in adolescents by using LCA and (b) to analyze the inter-class differences on the four components of aggression behavior (anger, hostility, physical aggression, and verbal aggression). Regarding the first aim, it is expected to find a similar perfectionism three-class solution in our sample of Spanish adolescents (i.e., high perfectionism, moderate perfectionism, and non-perfectionism) than that previously obtained by Vicent et al. (2019a) in a sample of Spanish children. Secondly, it is expected that those profiles defined by high levels of both perfectionism dimensions reported the highest mean scores on anger, hostility, physical aggression, and verbal aggression (Vicent et al., 2017).

## Materials and Methods

### Participants

A total of 1,074 high school students with an average socioeconomic level from various educational centers in the Region of Murcia participated in this study. The ages ranged from 12 to 18 years (*M* = 14.78, *SD* = 1.84). Selection of participants was random through a multi-stage cluster sampling, and clusters were geographical (urban, semi-urban, and rural), high schools (public and private), and classroom (randomly selected from compulsory education).

An initial sample of 1,724 participants was obtained. However, 650 students were excluded because questionnaires had missing data or errors, or because they were immigrant with low language domain, or because they did not assist to school when questionnaires were administered.

In the final sample, 60.1% were women, and 39.9% were men.

### Instruments

The Child-Adolescent Perfectionism Scale (CAPS) of Flett et al. (2016) was used, which is a 22-item instrument based on the multidimensional conceptualization of perfectionism (Hewitt and Flett, 2004). This instrument measures two subscales: Self-Oriented Perfectionism (e.g., “I get angry with myself when I make a mistake”), which was employed as an indicator of PS, and Socially Prescribed Perfectionism (e.g., “My teachers expect my work to be perfect”), which was employed as an indicator of PC. Participants were awarded 5-point grades for dealing with each item. The Spanish-translated version by Castro et al. (2004) was used in our study. This is a reliable instrument for research purposes (Vicent et al., 2017). In its original validation, the SPP obtained adequate indexes of internal consistency and test-retest reliability (α = 0.81; tr = 0.74). Castro et al. (2004) applied a version of the CAPS translated into Spanish, reporting acceptable levels of reliability and temporal stability for the Socially Prescribed Perfectionism and Self-Oriented Perfectionism that varied between α = 0.82 and 0.92 for a clinical and non-clinical sample of Spanish adolescent women. In this study, the Cronbach’s α coefficients were α = 0.72 for Self-Oriented Perfectionism and α = 0.75 for Socially Prescribed Perfectionism.

The Spanish version of the Aggression Questionnaire (AQ) developed by Santisteban and Alvarado (2009) was also used. Buss and Perry (1992) made the original version. The Spanish version was validated in pre-adolescent sample (age: 9–11 years). It consists of 29 items of a 5-point Likert (1 – uncharacteristic of me to 5 – very characteristic of me). This questionnaire provides a global measure of aggression and four subscales: physical aggression, with nine items; verbal aggression, with five items; anger, with seven items; and hostility, with eight items. This version obtained acceptable reliability indices for the four factors (Physical Aggression = 0.80, Verbal Aggression = 0.73, Anger = 0.65, and Hostility = 0.66) was used for this study. Examples of items per scale are: Physical Aggression (e.g., “If someone hits me, I respond by hitting him too”), Verbal Aggression (e.g., “When people do not agree with me, I cannot help arguing with them”), Anger (e.g., “Sometimes I feel like a bomb about to explode”), and Hostility (e. g., “When people are especially friendly, I wonder what they want”).

### Procedure

First, the authorization of the ethics committee for the development of the research was processed. Next, the high schools were selected. After the above, interviews were held with the directors and/or counselors of the educational centers to present the objectives, describe the evaluation instruments, request their permission, and promote their collaboration. In parallel, the authorization of the parents or legal representatives of the students and the consent of the students themselves were obtained. During the application session (lasting approximately 50 min) for evaluation of the instruments, the students were informed of the voluntariness, anonymity, and confidentiality of the study. The response templates were then coded and entered into a database for statistical treatment.

About 220 students did not take part in the study because they did not give informed consent or because their questionnaires were incomplete. Study participants participated in the study after informed written consent was obtained from both the minors and their parents.

### Data Analysis

A Latent Class Analysis (LCA) was used to identify the different groups of perfectionists. The best model was selected by analyzing the most appropriate values, i.e., the lowest values of the Bayesian Information Criteria (BIC) and entropy values close to 1 (Schreiber, 2017). The groups were defined according to their levels of PS and PC dimensions. ANOVA was used to examine the inter-class differences in the different manifestations of aggression. Cohen’s *d* test was calculated to estimate the magnitude of the differences (Cohen, 1998), considering small (between 0.20 and 0.49), moderate (between 0.50 and 0.79), and large effect sizes (higher than 0.80). Data were analyzed using the statistical package SPSS v22 and the Excel package XLSTAT to run the latent class analyses.

### Ethics Approval

The study protocols were approved by the Ethics Committee for Clinical Investigations of the University of Murcia (ID: 1405/2016). Moreover, this study was performed in accordance with the approved guidelines and the Declaration of Helsinki, with parental written informed consent obtained from all participants.

## Results

Table 1 outlines the Pearson’s correlations between the variables under study and the descriptive statistics (Mean and SD). There was a significant positive correlation between physical aggression and PC. Likewise, significant and positive correlations were found between verbal aggression, anger, hostility, PS, and PC. Therefore, it was relevant to proceed to the LCA.

**Table 1:** Pearson correlation between the variables of study and the descriptive statistics.

Variable	PS	PC	*M*	*SD*
Physical aggression	0.053	0.132^**^	20.21	7.17
Verbal aggression	0.080^**^	0.088^**^	11.28	3.90
Anger	0.182^**^	0.134^**^	17.84	5.01
Hostility	0.163^**^	0.203^**^	19.75	5.53
PS			39.46	6.40
PC			32.85	6.77

** *p* < 0.01.

The models obtained (from two to six classes) by LCA are shown in Table 2. Since a lower value of BIC and a higher entropy are the best indicators of the number of classes, among all the models, the model that had the most suitable BIC with the highest entropy values was selected, i.e., the 3-class model. This class solution was composed of three different perfectionism groups: (a) a first group of 717 students (66.8%) called Non-Perfectionists, since this group was characterized by low levels of both PC and PS; (b) a second group of 257 students (23.9%) called Maladaptive Perfectionists, characterized by high levels of PC and moderate levels of PS; and (c) a third group of 100 students (9.3%) called Adaptive Perfectionists, characterized by moderate scores in both perfectionism dimensions.

**Table 2:** The fits for the results of the latent class analysis of the study.

Account of classes	BIC	Entropy
2	8558.298	0.54
3	8452.512	0.59
4	8464.877	0.58
5	8468.985	0.55
6	8493.390	0.55

BIC, Bayesian Information Criterion.

Table 3 presents the results of the ANOVAs that revealed significant differences between classes. Different types of PC and PS showed statistically significant differences regarding the four components of aggression behavior.

**Table 3:** Mean scores, SDs, and *post hoc* contrasts obtained by the perfectionist classes on the different manifestations of aggression behavior.

	Non-Perfectionists		Maladaptive Perfectionists		Adaptive Perfectionists		Significance		
Variable	*M*	*SD*	*M*	*SD*	*M*	*SD*	*F_(2,1,071)_*	*p*	*η_p_^2^*
Physical aggression	20.11	7.05	21.38	7.50	17.95	6.60	8.62	<0.001	0.16
Verbal aggression	11.26	3.81	11.84	4.18	10.09	3.56	7.42	0.001	0.14
Anger	17.66	4.86	19.19	5.16	15.75	4.69	19.16	<0.001	0.35
Hostility	19.44	5.36	21.62	5.85	17.21	4.42	27.67	<0.001	0.49

In Table 4, *post hoc* comparisons revealed that Non-Perfectionists obtained significantly lower scores in physical aggression, anger, and hostility than Maladaptive Perfectionists. Non-Perfectionists obtained significantly higher scores in physical aggression, verbal aggression, anger, and hostility than Adaptive Perfectionists. Similarly, *post hoc* comparisons revealed that Maladaptive Perfectionists obtained significantly higher scores in physical aggression, verbal aggression, anger, and hostility than Adaptive Perfectionists. Small effect sizes were found for all differences with the exception of Adaptive vs. Maladaptive contrasts on anger and hostility which were of a moderate magnitude.

**Table 4:** Cohen’s *d* indexes for *post hoc* contrast groups.

Variable	Non-perfectionists vs. Maladaptive Perfectionists	Non-perfectionists vs. Adaptive Perfectionists	Adaptive Perfectionists vs. Maladaptive Perfectionists
Physical aggression	0.17^*^	0.31^*^	0.47^***^
Verbal aggression	n.s.	0.31^*^	0.44^***^
Anger	0.31^***^	0.39^**^	0.68^***^
Hostility	0.40^***^	0.42^**^	0.80^***^

* *p* < 0.05;

** *p* < 0.01;

*** *p* < 0.01.

## Discussion

This study allowed examining the existence of different classes of perfectionists according to their degree on the PS and PC dimensions: (a) a first group of 717 students, called Non-Perfectionists; (b) a second group of 257 students, called Maladaptive Perfectionists; and (c) a third group of 100 students, called Adaptive Perfectionists. Previous studies performed with techniques close to that employed in this study (i.e., LCA) also showed the existence of three clusters similar to those obtained in our study (Gilman et al., 2014; Moate et al., 2016; Wang et al., 2016). However, as it was expected, the three-class solution obtained in this study did not confirmed the four clusters identified by Vicent et al. (2017) which can be explained by the profile extraction method employed. Thus, as has been mentioned in the introduction section, Vicent et al. (2019b) used a non-hierarchical cluster analysis, whereas in this study, a more appropriate technique was employed (i.e., LCA).

On the other hand, the prevalence rates of the three classes identified in our study do not coincide with those of other previous studies, possibly due to the different instruments employed to assess perfectionism, as well as the sample characteristics such as cultural or age aspects (Non-Perfectionist = 66.7%; Maladaptive Perfectionist = 24%; and Adaptive Perfectionist = 9.3%).

Attending to the second aim of this study, significant differences were found in the different manifestations of aggression between the observed perfectionism classes. Thus, as expected consistently to Sherry et al. (2016), Maladaptive Perfectionists presented higher values in physical aggression, verbal aggression, anger, and hostility than Adaptive Perfectionists and Non-Perfectionists. It indicates that those adolescents with high values of both PC and PS (i.e., maladaptive perfectionists), can be more frequently involved in actions related to anger, hostility, or aggressiveness, either physical or verbal. On the other hand, Adaptive Perfectionists, with moderate levels on PS and PC, presented lower scores on the four components of aggression behavior in comparison with Non-Perfectionists. That is, young people classified as Non-Perfectionists presented a risk value in terms of aggression behavior, in comparison with Adaptive Perfectionists, so they need greater intervention to manage situations of anger, hostility, or physical and verbal aggression.

According to Hewitt et al. (2008), one of the core features of perfectionism is the need to be perfect. Researchers have also found that perfectionist self-presentation, defined as the need to appear as perfect in front of other, is associated with perfectionism dimensions of PS and PC (Hewitt et al., 2003). In addition, perfectionism is linked with several factors of psychological vulnerability, such as low self-esteem and symptoms of anxiety and depression (Hewitt et al., 1995, 2003). Therefore, this appearance of perfection in front of others can generate rejection and distancing from peers, leading to isolation, rejection, and victimization (Hewitt et al., 1995) which would be in line with the findings of this study.

In line with what was found in this study, there is great evidence in the literature on the relationship between PC and anger (Sherry et al., 2007; Hewitt and Flett, 2010; Saleh-Esfahani and Ali-Besharat, 2010). Some authors explain this relationship through frustration and the perception of humiliation felt by the subject. In the same way, there is agreement between the findings of this study and the previous literature regarding the relationship between hostility and PC. A person who is hostile and insecure will attribute his or her hostility to the environment. Thus, in a family environment, it has been suggested that perfectionists, due to their own evaluative concerns, may interpret any mild admonition from their parents as harsh criticism (Saleh-Esfahani and Ali-Besharat, 2010). Although there is less prior evidence for this, some authors, such as García-Fernández et al. (2017) and Öngen (2010), have shown a significant and positive relationship between PC and physical and verbal aggressiveness.

Regarding the relationship between PS and aggression behavior, Sherry et al. (2007) argued that individuals with high levels of PS are hypercompetitive, and this capacity can make them feel hostile toward others. In contrast, Stoeber et al. (2017) found that those with high PS may show more hostility, distrust, verbal aggression, and aggressive feelings, but it is their higher level of other oriented and socially prescribed types of perfectionism, which are responsible for these characteristics, not their higher levels of PS. In addition, PC generates social disconnection that may imply impaired interpersonal relationships (Sherry et al., 2016). However, according to our results, it seems that individuals characterized by high levels of both PS and PC dimensions reported the highest levels of aggression behavior. Even though there is a debate about the adaptive or the maladaptive nature of PS, most authors point out PC as a maladaptive construct (Sherry et al., 2016) which support it relation to different types of aggression. Hewitt et al. (2006) explain why perfectionism is associated with social disconnection and interpersonal hostility. They used three samples of college students (Ns = 318, 417, and 398) and completed measures of perfectionism and hostility (including aggression, anger, and spite). Their findings indicate that not all perfectionists feel socially disconnected and hostile toward others. Self-oriented perfectionists may feel socially connected and show no more hostility than non-perfectionists. In addition, as Dunkley and Blankstein (2000) suggest, PS are strongly correlated to PC that may be in the basis of their characterization of positive correlation between PS and PC dimensions and aggressive behavior.

In summary, this study has addressed the relationship between PC and PS with aggression behavior, considering its four components: anger, hostility, physical aggression, and verbal aggression.

One of the contributions of this study is its use of more powerful analysis tools, such as LCA, to confirm the existence of three perfectionism profiles (Adaptive Perfectionism, Maladaptive Perfectionism, and Non-Perfectionism) in Spanish adolescents. In addition, this study evidenced the relationship between the different profiles of perfectionism and aggression behavior, highlighting the strong need to work in schools to improve the social skills of subjects with perfectionist traits. Subjects characterized by high levels of PC will show higher physical and verbal aggression behaviors, incited by anger toward people who have imposed high standards on them, when said standards are not met. On the other hand, when these students are not able to satisfy the demands of others, given their PC, they will tend to perceive their environment as too critical and harsh. Thus, aggressive behaviors toward others can emerge in an attempt to defend themselves from an environment that they perceive as hostile (Vicent et al., 2019c). In addition, subjects characterized by high levels of PS showed competitive behaviors leading them to generate hostility toward others. This hostility can turn into anger or verbal aggression depending on the pressure that the subject feels in each situation to be the best (Vicent et al., 2017), although the reasons for PS and PC are diverse, both positively and significantly correlate with aggression behavior.

This study has some limitations. Firstly, it should be noted that the use of a cross-sectional design did not allow us to establish causal relationships and that longitudinal and experimental studies should be used to establish causal relationships between perfectionism and aggressiveness. Although this is a considerable sample in terms of its size, all our participants were recruited in the Region of Murcia, so it is not possible to generalize the results to the entire Spanish population. Other studies should be carried out with representative samples at the national or transnational level to allow generalization of the results. Furthermore, in this study, despite collecting gender data, no differential analyses were performed for this variable. It would be interesting to establish these differences in light of the other previous studies cited in this work.

## Conclusion

In conclusion, the results of this work show three to four profiles of adolescent perfectionism. Its relationship with aggressiveness is analyzed, distinguishing between physical and verbal aggressiveness, anger, and hostility. This work tries to overcome the limitations of the study by Vicent et al. (2017), trying to analyze the relationship between perfectionism and the four components of aggressiveness from a person-oriented approach, which contributes to greater knowledge on the subject.

Despite its limitations, the present work has several practical implications. Hence, social skills programs can improve school coexistence and may help deal with perfectionism, addressing both PS and PC facets. Therefore, future programs to prevent aggression in the school should consider including programs to reduce levels of perfectionism.

## Data Availability Statement

The raw data supporting the conclusions of this article will be made available by the authors, without undue reservation.

## Ethics Statement

The studies involving human participants were reviewed and approved by the University of Murcia, 1405/2016. Written informed consent to participate in this study was provided by the participants’ legal guardian/next of kin.

## Author Contributions

IM and CR-E: conceptualization, investigation, and data curation. IM and AF-S: methodology. IM and JA: formal analysis. All authors: writing – original draft preparation, writing – review and editing, and supervision. All authors contributed to the article and approved the submitted version.

### Conflict of Interest

The authors declare that the research was conducted in the absence of any commercial or financial relationships that could be construed as a potential conflict of interest.
